# PATIENT AND CARE PARTNER QUESTIONS AND CLINICIAN RESPONSES DURING INITIAL OUTPATIENT PALLIATIVE CARE APPOINTMENTS

**DOI:** 10.1093/geroni/igad104.3015

**Published:** 2023-12-21

**Authors:** Meghan McDarby, Hannah Silverstein, William Rosa, Patricia Parker, Brian Carpenter

**Affiliations:** Memorial Sloan Kettering Cancer Center, New York City, New York, United States; Washington University in St. Louis, St. Louis, Missouri, United States; Memorial Sloan Kettering Cancer Center, New York City, New York, United States; Memorial Sloan Kettering Cancer Center, New York City, New York, United States; Washington University in St. Louis, St. Louis, Missouri, United States

## Abstract

Person-centered palliative care (PC) requires person-centered communication. This study characterized questions asked by persons with neurologic illnesses and their care partners and PC clinicians’ responses. We analyzed audio recordings from 38 initial outpatient PC appointments to characterize questions stated by patients and care partners. Based on previous research, we identified two question types: direct questions (typically-phrased, stated to obtain or clarify information) and indirect questions (declarative statements, not phrased as a typical question, made with information seeking intent). We rated the completeness of clinicians’ responses on a 4-point scale ranging from 1 (completely addressed question and provided additional information) to 4 (no response). We also coded features of clinicians’ responses, including offers of support or action and unprompted recommendations. Patients and care partners asked both direct (n = 442; 79.6%) and indirect (n = 113; 20.4%) questions. More than 60% of all questions were about symptoms, treatment, and lifestyle issues. Clinicians responded to 90.9% of direct questions and 90.2% of indirect questions; their responses to both question types were similar in completeness (median = 1, mode = 1). Clinicians’ responses to direct questions included more offers of support or action compared to responses to indirect questions (17% v. 11%). Responses to indirect questions included more unprompted recommendations compared to responses to direct questions (21% v. 9%). Persons with neurologic illnesses and their care partners use different question types to obtain information during PC appointments. Implications include the need for clinician training to effectively respond to different question types and topics.

